# Map Building and Monte Carlo Localization Using Global Appearance of Omnidirectional Images

**DOI:** 10.3390/s101211468

**Published:** 2010-12-14

**Authors:** Luis Payá, Lorenzo Fernández, Arturo Gil, Oscar Reinoso

**Affiliations:** Departamento de Ingeniería de Sistemas Industriales, Universidad Miguel Hernández, Avda. de la Universidad s/n, 03202, Elche (Alicante), Spain; E-Mails: l.fernandez@umh.es (L.F.); arturo.gil@umh.es (A.G.); o.reinoso@umh.es (O.R.)

**Keywords:** global appearance, panoramic images, homomorphic filtering, Fourier Signature, topological mapping, monte-carlo localization

## Abstract

In this paper we deal with the problem of map building and localization of a mobile robot in an environment using the information provided by an omnidirectional vision sensor that is mounted on the robot. Our main objective consists of studying the feasibility of the techniques based in the global appearance of a set of omnidirectional images captured by this vision sensor to solve this problem. First, we study how to describe globally the visual information so that it represents correctly locations and the geometrical relationships between these locations. Then, we integrate this information using an approach based on a spring-mass-damper model, to create a topological map of the environment. Once the map is built, we propose the use of a Monte Carlo localization approach to estimate the most probable pose of the vision system and its trajectory within the map. We perform a comparison in terms of computational cost and error in localization. The experimental results we present have been obtained with real indoor omnidirectional images.

## 1. Introduction

Two well-known problems in mobile robotics are building a map of the environment where the robot moves and computing its location within this map. Finding a relatively good solution to both problems is crucial during the autonomous navigation of a mobile agent, which is expected to have to take decisions about its localization in the environment and about the trajectory to follow to arrive to the target points.

**Figure 1.**
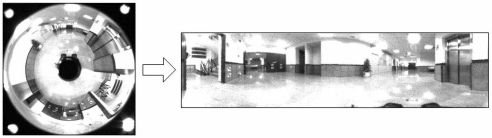
Omnidirectional and Panoramic scenes.

**Figure 2.**
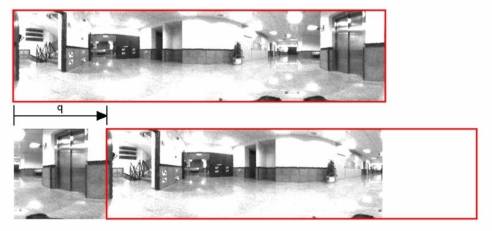
A robot rotation in the ground plane produces a shift in the columns of the panoramic images captured.

During the past years, omnidirectional cameras have become a widespread sensor in mobile robotics mapping and localization tasks, due to their low cost, weight and power consumption and to the richness of the information they provide us from the environment. In this work, we use the information captured by a camera that is installed at a fixed position on the robot and pointing upwards in direction to a hyperbolic mirror. This system offers us omnidirectional images from the environment. Different representations of the visual information can be used when working with these catadioptric systems (Figure 1), such as the omnidirectional, panoramic and bird-eye view images [1,2]. We use the panoramic representation of the scenes as it can offer invariance to ground-plane rotations when the movement of the robot is restricted to the ground plane. A pure rotation of the robot in the ground plane corresponds to a shift in the columns of the panoramic image (Figure 2).

Different authors have studied the use of omnidirectional images both in robot mapping and localization. These solutions can be categorized into two main groups: feature-based and appearance-based solutions. In the first approach, a number of significant points or regions are extracted from each omnidirectional image and each point is described using an invariant descriptor. As an example, Se *et al.* [3] carry out localization and mapping tasks using SIFT features [4,5] extracted from a set of images. Valgren and Lilienthal [6] and Murillo *et al.* [7] use the SURF features [8] extracted from omnidirectional images to find the location of the robot in a given map. On the other hand, the appearance-based approach works with the images as a whole, with no local feature extraction. Each image is represented by a single descriptor that contains information of its global appearance. As an example, Menegatti *et al* . [9] use the Discrete Fourier Transform (DFT) to build a visual memory from a set of panoramic images, and Menegatti *et al*. [10] perform a probabilistic localization within this memory. Kröse *et al*. [11] use Principal Components Analysis (PCA features) [12] of panoramic images for environment modeling and localization.

With respect to the mapping problem, the current research can be classified into two approaches: metric and topological. The first one consists of modeling the environment using a map obtained with geometrical accuracy when representing the position of the robot in the map. For example, Moravec and Elfes [13] describe a sonar-based mapping system developed for mobile robots navigation, Collins *et al*. [14] analyze the performance of several established mapping techniques using identical test data and Gil *et al*. [15] present an approach to carry out the mapping process with a team of mobile robots and visual information. On the other side, topological maps are graphical models of the environment that capture places and their connectivity in a compact form. An example of this method is presented by Werner *et al*. [16] where a topological map of the environment is obtained from a sequence of color histograms from visited places. Valgren and Lilienthal [17] study how to build a topological map of large indoor and outdoor environments using local features extracted from omnidirectional images and the epipolar constraint, and a clustering method to perform localization more efficiently. Stimec *et al*. [18] present an appearance-based method for path-based map learning by means of a clustering of the PCA features extracted from a set of panoramic images into distinctive visual aspects. At last, Tully *et al*. [19] present a probabilistic method for topological SLAM, solving the topological graph loop-closing problem by means of a tree expansion algorithm and Angeli *et al*. [20] develop an incremental topological mapping and localization approach, integrating metrical information from robot odometry.

The approach we use to carry out the mapping process is inspired by the work of Menegatti *et al*. [9]. Our main contribution is the incremental mapping process, which permits building the map online while the robot is traversing the environment. Other features that differentiate our work are (i) the use of homomorphic filtering to remove lighting effects in the images, (ii) the formalization of a method to describe the shape difference between the built map and the original grid, by means of removing the scale, rotation and reflection effects and (iii) the study of the dependence of the mapping process and the resulting map against the most interesting parameters of the process.

Once the map has been built, it is necessary to test if the robot is able to compute its pose (position and orientation) within the map with accuracy and robustness while knowing its location is crucial for an autonomous agent, since the pose is needed for a precise navigation. The *Monte Carlo Algorithm* has been extensively used in localization tasks in the field of mobile robotics, demonstrating robustness and efficiency [21]. Different approaches have been developed depending on the nature of the sensor installed on the robot. For example, Thrun *et al*. [22] use a laser range sensor, Dellaert *et al*. [23] a camera pointing to the ceiling and Gil *et al*. [24] a stereo camera. The information these systems provide is used to weight the particles and estimate the position of the robot. It is also possible to use external sensors to localize the robot, as Pizarro *et al*. [25] do with a single camera attached at a fixed place outside the robot. However, these approaches are not applicable to large configurations of the environment.

In this paper we propose to solve the localization problem using omnidirectional images and global appearance-based methods, as we do in the mapping task. Concerning the Monte Carlo localization methods using global appearance information, some similar works can be found in the literature, as the one of Menegatti *et al*. [10], who use Monte Carlo localization with Fourier Signatures as global image descriptors and path-based maps and mainly centers in the strategy to solve the robot kidnapping problem. In our approach, we use dense maps (grid-based maps) to carry out the experimentation and we propose and compare different weighting methods to optimize the localization task. In some of these weighting methods we have included some information from the orientation extracted from the omnidirectional images, which gives robustness to these approaches. We carry out the experimentation with different grid sizes in the map and different number of particles and all the results are decomposed in global localization and tracking. The main contribution of our work in this field consists of optimizing the parameters of the particle filter, as we show in the results section.

Another related work is presented by Linaker and Ishikawa [26]. They introduce PHLAC (Polar High-order Local Auto-Correlation) to describe the images in the global appearance domain, with an adaptation that makes it invariant against rotations when working with omnidirectional images. The work is focused in the study of the performance of the descriptor they introduce in a probabilistic Monte Carlo localization task, in a high perceptual aliasing situation and with noise and occlusions, using a relatively small environment (1.1 × 0.8 m size with a grid step of 0.1 m). We do not focus on the performance of the descriptor but in the parameters of the filter to optimize the localization process. We test in a variety of environments, from small ones to larger ones, with a maximum step of 1*m*. As far as other vision-based probabilistic localization works are concerned, they focus mainly in the extraction of significant points to carry out the localization process.

So, in this work, we focus on the analysis of different choices that permit building topological maps from a set of omnidirectional images, so that localization can be carried out within these maps. Our main objective consists of evaluating the feasibility of using purely global-appearance methods in these tasks. However, solving the SLAM problem and studying the features of the appearance descriptor is out of the scope of this work. This way, we present first a methodology to built a grid map of the environment using an incremental method, with several omnidirectional images captured along the environment. We use a mass-spring-damper model with this aim. The method is able to arrange these images to create a topological map whose layout is similar to the layout of the grid where the images were captured. Also, in a second phase, we propose different methods that allow us to localize the robot using a particle filter and new methods to refine the position of the robot rapidly. We have decided to describe each omnidirectional image by a single Fourier descriptor. However, the methods described here are in fact independent of the descriptor used to represent the images, and other appearance-based descriptors may also be applied.

The work is structured as follows. Section 2 presents the fundamentals of global appearance-based approaches and the processing on the images to obtain a robust descriptor to make the experiments. Section 3 shows the description of a method for batch topological mapping and a new technique for building topological maps incrementally. Section 4 deals with the Monte Carlo algorithm and its application to the problem of localization in mobile robotics using the appearance of omnidirectional images. In this section we describe the different types of weight employed to make the localization experiments. Next, Section 5 presents the results of the mapping and localization experiments. All these experiments have been carried out using a real robotic platform and several sets of omnidirectional images captured with this platform. Finally, we present the conclusions and future work in Section 6.

## 2. Appearance-based Techniques

In this section we present the state of the art and the procedure we have followed to build the global descriptor of each omnidirectional image. These descriptors have been previously used by other authors [9].

### 2.1. State of the art

The appearance-based techniques offer a systematic and intuitive way to construct the map and carry out the localization process. With these techniques, each image is described by means of a single global descriptor and the information from these descriptors is used to build the map (*i.e.*, to establish relationships between locations) and to carry out the localization process. These techniques are especially useful when the robot moves in an unstructured environment, where the creation of appropriate models of recognition can be a difficult task. However, as no relevant information is extracted from the images, it is usually necessary to apply a compression technique to reduce the computational cost of the mapping and localization processes.

PCA (Principal Components Analysis) is a widely extended method used to extract the most relevant information from a set of images [11]. However, the main problem of PCA methods is that they are not inherently invariant to the ground-plane orientation of the robot. Some authors have showed how to build an appearance-based map of an environment using a variation of PCA that includes information not only about the localizations where the images were taken but also about the possible orientations at that points [27]. However all the images must be available before building the PCA descriptors, and if a new image must be added to the map, all the descriptors must be computed from scratch or by means of a computationally quite heavy process.

Other researchers rely on DFT (Discrete Fourier Transform) methods to get the most relevant information from the images. In this case there are several possibilities, such as to implement the 2D Discrete Fourier Transform [1], the Spherical Fourier Transform of omnidirectional images [28] or the Fourier Signature of the panoramic image [9]. In the three cases, the descriptor presents rotational ground-plane invariance and concentrates the most relevant information in the low frequency components of the transformed image. Also, each image descriptor is computed independently of the rest of images.

Based on a previous work [1], we have decided to make use of the Fourier signature to build the image descriptors. The processing time needed to compute the Fourier Transform is substantially lower than in common feature extraction and description methods and it allows a fast comparison between the current image and the map by means of a vector distance measurement.

Due the fact that the appearance of an image depends strongly on the lighting conditions of the environment to map [29], the descriptor must be robust against these lighting changes. To avoid the problems of illumination variation, some proposals can be found in the literature. Murase and Nayar [30] make use of an appearance-based approach and solve the problem by generating many views of the object under different lighting conditions. Other researchers make use of edge-detection filters or homomorphic filtering to separate the components of luminance and reflectance [31]. In a previous work [32] we have proved that when applying a bank of homomorphic filters on the images, it is possible to minimize the dependence with respect to the lighting conditions of the environment to map.

### 2.2. Global appearance descriptor

In this section we present the methodology we have followed to describe the appearance of the scenes in an efficient and robust way. This descriptor must be robust against small changes in the environmental lighting conditions, the computational cost to obtain it must be low to allow mapping and localization in real time, and it has to be built in an incremental way so that it allows the map to be created while the robot is going through the environment (*i.e.*, the descriptor of an image must not depend neither on the previous nor on the later captured images). We build our descriptor using the Fourier Signature over a set of previously filtered omnidirectional images.

#### Fourier Signature with panoramic images

Among the Fourier-based methods, the advantages of the Fourier signature are its simplicity, computational cost and the fact that it exploits better the invariance against ground-plane rotations using panoramic images. When we have an image *I_j_* with *N_x_* rows and *N_y_* columns, we can obtain the most relevant information from the image by means of the Discrete Fourier Transform. To compute the Fourier signature we have to expand each row of the panoramic image {*a_n_*} = {*a*_0_, *a*_1_, . . ., *a*_*N_y_*−1_} using the Discrete Fourier Transform into the sequence of complex numbers {*A_n_*} = {*A*_0_, *A*_1_, . . ., *A*_*N_y_*−1_}.

This Fourier signature presents the same properties as the 2D Fourier Transform. The most important information is concentrated in the low frequency components of each row, so we can work only with the information from the *k* first columns in the signature (*k* < *N_y_*), and it presents rotational invariance when working with panoramic images. It is possible to prove that if each row of the original image is represented by the sequence {*a_n_*} and each row of the rotated image by {*a_n_*_−_*_q_*} (being *q* the amount of shift) (Figure 2), when the Fourier Transform of the shifted sequence is computed, we obtain the same amplitudes *A_k_* than in the non-shifted sequence, and there is only a phase change, proportional to the amount of shift *q*, (Equation 1).

(1) $$F\left[\left\{a_{n-q}\right\}\right]=A_{k}\text{exp}\left(-j\frac{2\pi ql}{N_{y}}\right);\;\;\;\;\;l=0,\;\ldots \;,N_{y}-1$$

We can separate the computation of the robot position and the orientation thanks to this shift theorem. Finally, it is interesting to highlight also that the Fourier Signature is an inherently incremental method.

To compute the difference between the appearance of two scenes, we use the Euclidean distance between the Fourier signature. If the bi-dimensional vector *d_i_*(*u, v*), with size *N_x_* × *k* is the Fourier signature of the image *I_i_*(*x, y*) and *d_j_*(*u, v*), with size *N_x_* × *k* is the Fourier signature of the image *I_j_*(*x, y*), then the distance between scenes *i* and *j* is:

(2) $$D_{ij}=\sqrt{\sum_{u=0}^{N_{x}}\sum_{v=0}^{k}{\left(d_{i}(u,\;v)-d_{j}(u,\;v)\right)}^{2}}$$

#### Homomorphic filtering

When the mobile robot moves in a real environment, it has to cope with some typical situations that produce small changes in the appearance of the environment, such as the lighting conditions and changes in the position of some objects. This way, the descriptor built must be robust against these small variations in the environment.

There are different methodologies to provide robustness to the map created. In a previous work [32], we show how it is possible to increase the accuracy when locating a robot in a previously created map applying Homomorphic filtering techniques on the panoramic images captured. In this work, we have used such techniques to filter the images as a preprocessing step before extracting the Fourier signature.

The homomorphic filtering allows us to filter separately the luminance and reflectance components of an image [33]. Thus, we can control the influence of each component on the image appearance. The homomorphic filter uses the natural logarithm operator on the image to separate the components of luminance and reflectance:

(3) $$\begin{array}{r} I(x,\;y)=l(x,\;y)\times r(x,\;y)\\ z(x,\;y)=\text{ln}(I(x,\;y))\\ z(x,\;y)=\text{ln}(l(x,\;y))+\text{ln}(r(x,\;y)) \end{array}$$

where *I*(*x, y*) corresponds to the panoramic image and can be expressed as a multiplication of the luminance *l*(*x, y*) and the reflectance component *r*(*x, y*) of the image. When the components have been separated, we can apply a filter on the image in the frequency domain. Previously, it is necessary to apply the 2D Discrete Fourier Transform on the panoramic image:

(4) $$\begin{array}{r} F[\{z(x,y)\}]=F[\{\text{ln}(l(x,\;y))\}]+F[\{\text{ln}(r(x,\;y))\}]\\ F[\{z^{\prime }(x,\;y)\}]=F[z\{x,\;y\}]\cdot H(u,\;v) \end{array}$$

where *H*(*u, v*) is the filter transfer function in the frequency domain. The low frequency components are associated with the illumination of the image and the high frequency ones with the reflectance. To reduce the effects of changes in the lighting of the scenes, we apply a high pass filter constructed from a Butterworth low pass filter [33]:

(5) $$\begin{array}{r} D(u,\;v)={\left(u^{2}+v^{2}\right)}^{\frac{1}{2}}\\ H_{lp}(u,\;v)=\frac{1}{1+{\left[\frac{D(u,\;v)}{D_{0}}\right]}^{2n}}\\ H_{\text{hp}}^{\prime }(u,\;v)=1-H_{lp}(u,\;v)\\ H_{hp}(u,\;v)=(\alpha_{h}-\alpha_{l})\cdot H_{hp}^{\prime }(u,\;v)+\alpha_{l} \end{array}$$

where *D*(*u, v*) is the distance to the origin in the frequency domain, *D*_0_ is the filter cut-off frequency to construct the low pass filter, *n* is the order of the filter and *H_lp_*(*u, v*) is the low pass filter transfer function in the frequency domain. The last two expressions are used to build the high pass filter from the low pass filter, where *α_h_* and *α_l_* correspond to the maximum and minimum value of the homomorphic filter and *H_hp_*(*u, v*) is the high pass filter transfer function in the frequency domain. When working with homomorphic filters, the parameters of the filter need to be adjusted previously. Further information about the optimal values for the parameters can be found in [32].

## 3. Topological Map Building

**Figure 3.**
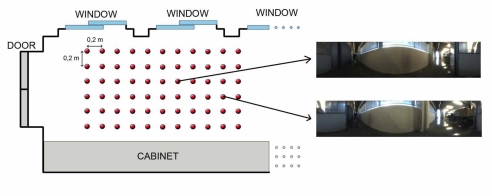
Bird eye’s view of the *corridor 1* environment grid where omnidirectional images have been taken to perform map building and localization.

**Figure 4.**
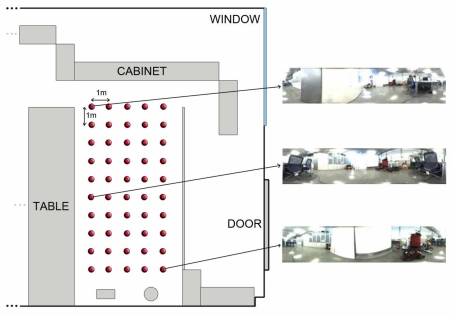
Bird eye’s view of the *laboratory 1* environment grid.

Once the kind of information to store in the database has been studied, we need to establish some geometrical relationships between the stored locations to complete the mapping process. In other words, the set of scenes the robot has captured along the environment must be arranged so that the resulting map has a similar layout comparing to the actual layout where the images were captured. Several databases with images from different environments have been used to test the algorithms. All of them consist of a set of omnidirectional images captured on a regular grid. The main features of these databases are shown in table 1. As an example of the these databases, we show in Figure 3 a bird eye’s view of the *corridor 1* database, with the grid points where the set of omnidirectional images have been captured and two examples of these images. This environment is a corridor with 2.4 × 1.5 m size and 0.2 m grid step (distance between the points where images were captured) and it presents a high degree of perceptual aliasing. In Figure 4 we show the *laboratory 1* database, a laboratory with 6 × 11 m size and 1 m grid step.

### 3.1. Mass-spring-damper model

To build a topological map of the environment where the images’ layout matches up with the original layout, we make use of a mass-spring-damper model, where each image acts as a particle *P_i_, i* ∈ {1, . . ., *n*} whose mass is *m_i_*. Each pair of particles *P_i_* and *P_j_* are joined with a spring *S_ij_* with elastic constant *k_ij_* and a damper with damping constant *κ_ij_*. If the value of the elastic constants is proportional to the distance between images, then when this particle system freely evolves to the balance, it is expected the final layout of the particles is similar to the real layout where images were captured.

The strength acting over each particle *i* of the system is:

(6) $${\vec{F}}_{i}=\sum_{i,\;j=\{1,\ldots ,n\},\;\;\;i\neq j}\left(-k_{ij}\cdot (l_{ij0}-l_{ij})-k_{ij}\cdot \left\|{\vec{v}}_{i}-{\vec{v}}_{j}\right\|\right)$$

where *l_ij_*_0_ is the natural length of the *S_ij_* spring, *l_ij_* is the current length of this spring, and ${\vec{v}}_{i}$ and ${\vec{v}}_{j}$ are the velocities of the particles *P_i_* and *P_j_*. In our experiments, we make *l_ij_*_0_ = *D_ij_* (Equation 2), *i.e.*, the distance between the Fourier signature of images *i* and *j*.

Taking into account the resulting force on each particle, we can update the position of this particle at each iteration with the following expressions:

(7) $$\begin{array}{r} {\vec{a}}_{i}={\vec{F}}_{i}/{\vec{m}}_{i}\\ {\vec{v}}_{i}(t+\Delta T)={\vec{v}}_{i}(t)+{\vec{a}}_{i}(t)\cdot \Delta T\\ {\vec{r}}_{i}(t+\Delta T)={\vec{r}}_{i}(t)+{\vec{v}}_{i}(t)\cdot \Delta T \end{array}$$

where ${\vec{a}}_{i}$, ${\vec{v}}_{i}$ and ${\vec{r}}_{i}$ are the acceleration, velocity and position of the *i*th particle, whose mass is *m_i_*. In our experiments, all the particles have been assigned the same mass (*m_i_* = 1 ∀*i* ∈ {1, . . ., *n*}). The parameter Δ*T* that appears in these equations has a significant relevance in the necessary time to tend to balance and in the accuracy of the final particle distribution. It is desirable that this parameter take a relatively high value during the first iterations, when the particles are far from the balance and they must move perceptively. As the system tends to balance, this parameter should take a lower value due to the fact that particles are expected to be closer to its final position as the process advances. In our experiments, we have set a maximum number of steps *stot* and we make Δ*T* dependent on it:

(8) $$\Delta T=\xi \cdot \left(1-\frac{s}{s_{\text{tot}}}\right)$$

**Figure 5.**
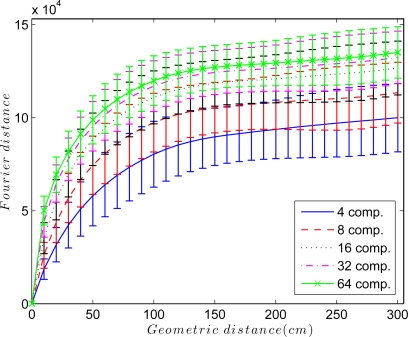
Euclidean distance between the Fourier signatures versus geometrical distance between the points where the images were captured.

An interesting parameter to adjust is the elastic constant *k_ij_* of each spring in the system. Figure 5 shows the Euclidean distance between the Fourier signatures versus the geometrical distance between the points where the images were captured retaining *k* = 4, 8, 16, 32 and 64 Fourier components per row. In all cases, the Fourier distance behaves approximately linear in the surroundings of the point where the image was captured. This linearity disappears as we move away from it, and the distance may even decrease. To solve this problem, the elastic constants take a value that depends on the Euclidean distance between Fourier signatures *D_ij_* and the components per row we retain in the Fourier Signature *k*, so *k_ij_* = *f*(*D_ij_, k*). Taking this fact into account, to build the map, only the nearest images to each one are taken into account in the force system, *i.e.*, only the images below a distance threshold are linked with springs.

On the other hand, the damping constants *κ_ij_* have an important role in the convergence of the mapping process. Thanks to them, the update of the system at each iteration is not a sudden process and the system tends more gradually to balance.

The mapping process is strongly conditioned by the availability of the omnidirectional images along this process. When all the images are available before the mapping process begins, a batch mapping approach must be used. However, if the images are captured gradually and the mapping process must start before all the images are available, an incremental approach must be implemented where the map is updated when a new image is captured. These two approaches are addressed in the next subsections.

### 3.2. Batch topological mapping

When we have a set of panoramic images from an environment, with no information about the order they were acquired in, a batch approach must be used to build the topological map. In this approach, all the particles are given a random initial position. Then, we leave the system evolve by successively applying Equations 6–8. The system is considered to have arrived to balance when the sum of movements of all the particles is under a threshold.

This approach may become computationally unfeasible for large environments where a big collection of images has been captured. The computational cost grows exponentially with the number of images, due to the increasing number of forces on each particle. Also, it must be taken into account that all the images must be available before starting the process so it is not possible to perform an online mapping process (where the map must be built as the explorer robot goes through it).

**Figure 6.**
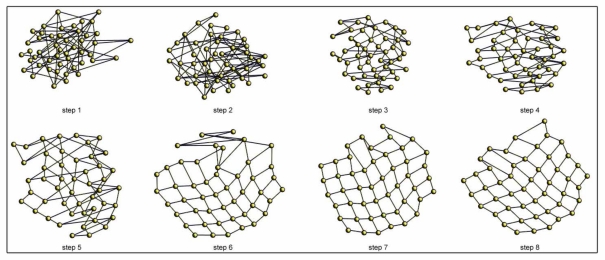
Example of some intermediate steps during the batch mapping process of the *office* environment (6 *×* 8 images grid).

Figure 6 shows an example of the batch topological mapping process. We show some intermediate steps until the balance is reached for the *office* environment (Table 1). In this case, all the particles (images) are present in the system from the first iteration.

### 3.3. Incremental topological mapping

This approach exploits the fact that during the mapping process the storage order of the images is usually known. Thanks to the incremental approach, the map can be built online, and when a new image is captured, the map is updated according to it. As the information is added gradually, the process becomes more robust and computationally more efficient. When we have a balanced set of particles *P_i_, i* ∈ {1, . . ., *m*}, when a new particle *P_m_*_+1_ arrives, we allow this particle to tend to balance (using Equations 6–8) while maintaining the position of the other particles fixed. Once the movement of the particle is under a threshold, we allow the whole system to tend to balance. This process compensates the singularities and false minima that the new particle produces over the whole system. We can avoid giving the new particle *P_m_*_+1_ a random initial position as it can be approximately inferred from the position of the previous particle in the system, *P_m_*. This would facilitate the convergence of the system.

**Figure 7.**
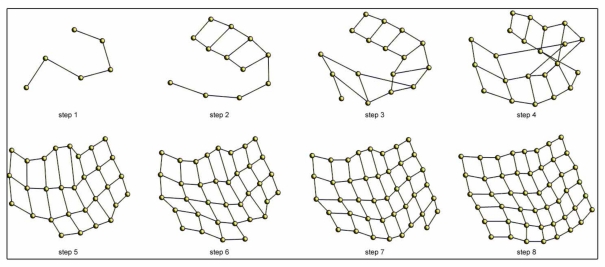
Example of some intermediate steps during the incremental mapping process of the *office* environment (6 *×* 8 images grid).

Figure 7 shows an example of the incremental topological mapping process. We show some intermediate steps until the balance is reached for the *office* environment (Table 1). The process starts with one particle, and a new one is added at each iteration.

### 3.4. Shape difference between the original and the resulting map layout

Once the map is built, we need a mechanism to evaluate how similar is the layout of the resulting map comparing to the real layout of the captures. As the only information we have used to build the map is the distance between Fourier signatures, the resulting map is expected to have a similar shape comparing to the original grid but with a scale factor, a rotation and a possible reflection. These effects should be removed in the resulting map to get an acceptable measure of the shape difference between the original and the resulting map layout. With this aim, we use a method based on the Procrustes analysis. This method is a statistical shape analysis that can be used to evaluate shape correspondence [34]. First, we arrange the coordinates of the points that form the grid were the images were captured in a matrix *A* = [(*α*_1_, *β*_1_)*^T^*, (*α*_2_, *β*_2_)*^T^*, . . ., (*α_n_*, *β_n_*)*^T^*]*^T^* and we arrange the coordinates of the balanced particles of the spring-mass-system in a matrix *C* = [(*γ*_1_, *δ*_1_)*^T^*, (*γ*_2_, *δ*_2_)*^T^*, . . ., (*γ_n_*, *δ_n_*)*^T^*]*^T^*. The Procrustes analysis allows us to compare the shape of these two grids by determining a linear transformation (a translation *c*, a reflection, an orthogonal rotation *T* and a scaling *b*) of the points in the matrix *C* so that the points in *b* · *C* · *T* + *c* to best conform to the points in the matrix *A*.

Once the translation, rotation and scaling effects have been removed, the goodness of fit criterion is the sum of squared errors. Thanks to this analysis we can measure how accurate is the layout of the particles after the mapping process, comparing to the layout where the images were taken.

This analysis has a closed form, as detailed in [34]. As a result of this process, a parameter *μ* ∈ [0, 1] can be obtained. *μ* is a measure of the shape correspondence between the sets of points *A* and *C*. The lower is *μ*, the more similar are *A* and *C*. We name this parameter “shape difference” along the paper. This shape difference is used in this paper with the only purpose to know the feasibility of the spring-mass system mapping model, and its use is possible due to the fact that we know the coordinates of the points in the original grid.

## 4. Monte Carlo Localization

In mobile robot localization we are interested in the estimation of the robot’s pose (location and orientation, typically, the state *x_t_* = (x, y, θ)) at time *t* using a set of measurements *z*_1:_*_t_* = {*z*_1_*, z*_2_*, . . ., z_t_*} from the environment and the movements *u*_1:_*_t_* = {*u*_1_*, u*_2_*, . . ., u_t_*} of the robot [35]. In *Monte Carlo Localization (MCL)* [21], the probability density function *p*(*x_t_|z*_1:_*_t_, u*_1:_*_t_*) is represented by a set of *M* random samples $\chi_{t}=\{x_{t}^{i},\;i=1\ldots M\}$ extracted from it, named particles. Each particle can be understood as a hypothesis of the true state of the robot $x_{t}^{i}=(x^{i},\;y^{i},\;\theta^{i})$. The weight of each sample (particle) determines the importance of the particle. The set of samples defines a discrete probability function that approximates the continuous belief.

### 4.1. Monte Carlo Localization algorithm

The initial set of particles represents the initial knowledge *p*(*x*_0_) about the state of the mobile robot on the map. When we use a particle filter algorithm, in global localization, the initial belief is a set of poses drawn according to a uniform distribution over the robot’s map. If the initial pose is partially known up to some small margin of error (local localization or tracking), the initial belief is represented by a set of samples drawn from a narrow Gaussian centered at the known starting pose of the mobile robot. The *Monte Carlo Localization algorithm* is described briefly in the next lines, and consists of two phases:

**Prediction Phase**: At time *t* a set of particles $\bar{\chi_{t}}$ is generated based on the set of particles *χ_t−_*_1_ and a control signal *u_t_*. This step uses the motion model *p*(*x_t_|x_t−_*_1_*, u_t_*). In order to represent this probability function, the movement *u_t_* is applied to each particle while adding a pre-defined quantity of noise. As a result, the new set of particles $\bar{\chi_{t}}$ represents the density *p*(*x_t_|z*_1:_*_t−_*_1_*, u*_1:_*_t_*).

**Update Phase**: In this second phase, for each particle in the set $\bar{\chi_{t}}$, the observation *z_t_* obtained by the robot is used to compute a weight $w_{t}^{i}$. This weight represents the observation model *p*(*z_t_|x_t_*) and is computed as $w_{t}^{i}=p(z_{t}|x_{t}^{i})$. In the following subsection we propose different methods for the computation of this weight. The weights are normalized so that $\sum w_{t}^{i}=1$. As a result, a set of particles accompanied by a weight $\bar{\chi_{t}}=\{x_{t}^{i},\;w_{t}^{i}\}$ are obtained.

The resulting set *χ_t_* is calculated by resampling with replacement from the set *χ̄_t_*, where the probability of resampling each particle is proportional to its importance weight $w_{t}^{i}$, in accordance with the literature on the SIR algorithm (Sampling Importance Resampling) [36,37]. Finally, the distribution *p*(*x_t_|z*_1:_*_t_, u*_1:_*_t_*) is represented by the set *χ_t_*.

### 4.2. Weight methods

By means of computing a weight *w^i^* for each particle and performing a resampling process, the Monte Carlo algorithm introduces the current observation *z^t^* of the robot. In this case we consider that our map is composed of a set of *N* bi-dimensional landmarks *L* = {*l*_1_*, l*_2_*, . . ., l_N_*} and the position of these marks on the environment is known. These landmarks form a grid in the environment with a particular resolution. Each landmark *l_j_* is represented by an omnidirectional image *I_j_* associated and a Fourier descriptor *d_j_* that describes the global appearance of the omnidirectional image, thus *l_j_* = {(*l_j,_*_x_*, l_j,_*_y_)*, d_j_, I_j_*}. *d_j_* is constructed from the bi-dimensional Fourier signature with all the elements arranged in a vector.

We consider that the robot captured an image at time *t* and computed the Fourier descriptor *d_t_*. Using this Fourier descriptor we compare the descriptor *d_t_* with the rest of descriptors *d_j_*, *j* = 1 . . . *N* and find the *B* landmarks in the map that are closest in appearance with the current image *I_t_*. In this sense, we allow the correspondence of the current observation to several landmarks in the map. We consider that this is a special case of the data association problem. In addition, this correspondence benefits the localization algorithm, since it may restrict the computation of the observation model to a reduced set of landmarks, thus reducing the computational effort. We will show results when varying this parameter in order to assess its influence. In addition, the selection of *B* landmarks in terms of appearance will allow us to evaluate the importance of the description method used.

To carry out the localization process we have used the Monte Carlo algorithm explained in the previous subsection. The computation of the particle weight is a very important part of the Monte Carlo algorithm. We propose several methods that allow us to compute the weight of each particle $w_{t}^{i}=p(z_{t}|x_{t}^{i})$, thus providing different observation models:

- Particle Weight Method 1 (PW1): This method correspond with a sum of gaussians centered on each image landmark, considering the difference in the descriptors.
  (9) $$w_{t}^{i}=\sum_{j=1}^{B}\text{exp}\{-v_{j}\sum_{l}^{-1}v_{j}^{T}\}\text{exp}\{-h_{j}\sum_{d}^{-1}h_{j}^{T}\}$$
  where, *v_j_* = (*l_j,_*_x_*, l_j,_*_y_) − (x*^i^*, y*^i^*) is the difference between the position of the landmark *l_j_* and the position (x*^i^,* y*^i^*) of the particle *i*. The matrix Σ*_l_* is a diagonal matrix $\sum_{l}=\text{diag}(\sigma_{l}^{2},\;\sigma_{l}^{2})$. The variance $\sigma_{l}^{2}$ is chosen experimentally in order to minimize the error in the localization. *h_j_* = *|d_j_* *− d_t_|* defines the difference between the module of the Fourier descriptor associated to the current image observed and the module of the descriptor associated to the landmark *l_j_*. The descriptors are normalized so that the summation of the euclidean distance of the current descriptor *d_t_* to the rest of the *B* associations equals one, $\sum_{j=1}^{B}h_{j}=1$. The matrix $\sum_{d}=\text{diag}(\sigma_{d}^{2})$ is a *k* × *k* matrix, being *k* the length of the Fourier descriptor. In this case, the observation model *p*(*z_t_|x_t_*) is not gaussian, since it is formed by a sum of gaussians, thus being multi-modal. This fact generally gives higher weights to particles situated near a landmark that is close in appearance to the current observation.
- Particle Weight Method 2 (PW2): Unlike the previous method, this method consists of a product of gaussians centered on each image landmark, considering the difference in the descriptors.
  (10) $$w_{t}^{i}=\prod_{j=1}^{B}\text{exp}\{-v_{i}\sum_{l}^{-1}v_{j}^{T}\}\text{exp}\{-h_{j}\sum_{d}^{-1}h_{j}^{T}\}$$
  Where *v_j_*, *h_j_*, Σ*_l_* and Σ*_d_* have been defined in the previous method. We recall that the product of gaussian distributions is also gaussian. The results demonstrate that this method tends to center the particles rapidly near the true pose, however, it suffers from some problems when the data association phase fails (e.g., the selected landmark *l_j_* lies far away from the actual robot pose).
- Particle Weight Method 3 (PW3): In this case, we have used a sum of gaussians centered on each landmark position and considering the difference in the descriptors as well as the orientation of the landmarks (images)
  $$w_{t}^{i}=\sum_{j=1}^{B}\text{exp}\{-v_{j}\sum_{l}^{-1}v_{j}^{T}\}\cdot \text{exp}\{-h_{j}\sum_{d}^{-1}h_{j}^{T}\}\cdot \text{exp}\{-g_{j}\sum_{\theta }^{-1}g_{j}^{T}\}$$
  where *v_j_*, *h_j_*, Σ*_l_* and Σ*_d_* have been defined in the previous methods. The variable *g_j_* = (*θ_j_* − *θ_i_*) computes the difference between the expected orientation *θ_j_* and *θ_i_* the orientation of the particle. Given the current descriptor *d_t_* and the descriptor *d_j_* the orientation *θ_j_* can be computed as in Equation (1). In this case, and since the map is known, the orientation of all the landmarks (images) in the map is known in advance. The matrix Σ*_θ_* is selected experimentally.
- Particle Weight Method 4 (PW4): This weight method is a product of gaussians centered on each landmark position and considering the difference in the descriptors as well as the orientation of the landmarks (images).
  (11) $$w_{t}^{i}=\prod_{j=1}^{B}\text{exp}\{-v_{j}\sum_{l}^{-1}v_{j}^{T}\}\cdot \text{exp}\{-h_{j}\sum_{d}^{-1}h_{j}^{T}\}\cdot \text{exp}\left\{-g_{i}\sum_{\theta }^{-1}g_{j}^{T}\right\}$$
  Where *v_j_*, *h_j_*, *g_j_*, Σ*_l_*, Σ*_d_* and Σ*_θ_* have been defined in the previous methods. This method is similar to the PW3, but considering the product of the gaussian distributions.
- Particle Weight Method 5 (PW5): In this case we use a gaussian distribution at the mass center of a discrete mass system. This method is inspired in a masses system, each one having a mass related to the similarity with the current descriptor *d_t_* observed by the robot. The weight for each mass is computed as:
  (12) $$w_{t}^{i}=\text{exp}\left\{-f_{j}\sum_{f}^{-1}f_{j}^{T}\right\}$$
  where *f_j_* = ((x*^i^,* y*^i^*) − *ĉ*) computes the difference between the position of the mass *i* and the mass center computed as:
  (13) $$\hat{c}=\sum_{j=1}^{B}l_{j}\cdot m_{j}$$
  where the virtual mass *m_j_* is computed as $m_{j}=\text{exp}\{-h_{j}\sum_{d}^{-1}h_{j}^{T}\}$. The masses *m_j_* are normalized so that $\sum_{j=1}^{B}m_{j}=1$ and the covariance matrix Σ*_f_* is computed as the covariance associated to *ĉ*.
- Particle Weight Method 6 (PW6): As in the previous one, this method uses a gaussian distribution at the center of a mass system, but in this case, the method obtains this center from a spring-mass system. The elastic constant of each spring is related to the similarity in the description, thus, landmarks more similar to the current observation try to attract the mass more tightly. To simplify the calculations, *m_j_* is equal to 1 for all the masses of the system. To compute the weight for each particle we use the following equation:
  (14) $$w_{t}^{i}=\text{exp}\{-f_{i}\sum_{s}f_{j}^{T}\}$$
  where *f_j_* = ((x*^i^,* y*^i^*) − *c̄*) computes the difference between the position of the particle *i* and the center *c̄* of a spring-mass system. In this case, the matrix Σ*_s_* is computed as the covariance associated to *c̄*.
- Particle Weight Method 7 (PW7): This weight method consists of a triangular distribution and is inspired in the weight function introduced by [10]. The weight for each particle is computed as:
  (15) $$w_{t}^{i}=\frac{1}{B}\sum_{j=1}^{B}S_{j}(D_{\text{max}}^{i}-\sqrt{v_{j}v_{j}^{T}})$$
  where $S_{j}=(1-\sqrt{h_{j}h_{j}^{T}})$ and *v_j_* = (*l_j,_*_x_*, l_j,_*_y_) − (x*^i^,* y*^i^*) computes the difference between the position of the particle *i* and the landmark *j*. $D_{\text{max}}^{i}$ is the metric distance between the farthest landmark and the position of the particle *i*. This weight method represents a triangular distribution centered on each landmark as to the appearance of each acquired image.

## 5. Results and Discussion

We have designed a complete set of experiments in order to test the validity of the global appearance-based approach both in map building and in localization.

### 5.1. Map building experiments

First, we have developed some experiments to know the accuracy and feasibility of the appearance-based approaches in map building. With this aim, we use several sets of omnidirectional images that have been captured in different structured and non-structured indoor environments (Table 1).

Our main objective consists of testing the feasibility of the batch and the incremental mapping procedures exposed in Section 3. With this aim, we study some features that define the feasibility of the procedures, such as the accuracy of the map built (how similar it is comparing to the grid where the images were captured) and the computational cost of the method. We also study how these features are influenced by some typical parameters, such as the number of images, the distance between them (grid step), the degree of compression during the Fourier Transformation and the value of the parameters of the mass-spring-damper model.

The preprocessing of the omnidirectional images includes the transformation to panoramic, homomorphic filtering and Fourier signature calculation. Once all the Fourier signatures of the images sets are available, the spring-mass-damper procedure is applied (Equations 6–8). In our experiments, the length of each spring *l_ij_* has been computed as the Euclidean distance between the main harmonics in the Fourier signature of images *i* and *j* (Equations 2). It is desirable to retain only the low-frequency components in the Fourier signature, where the main information is concentrated. In our experiments, we have worked with *k* = 4, 8, 16, 32 and 64 components per row in the Fourier signature.

**Figure 8.**
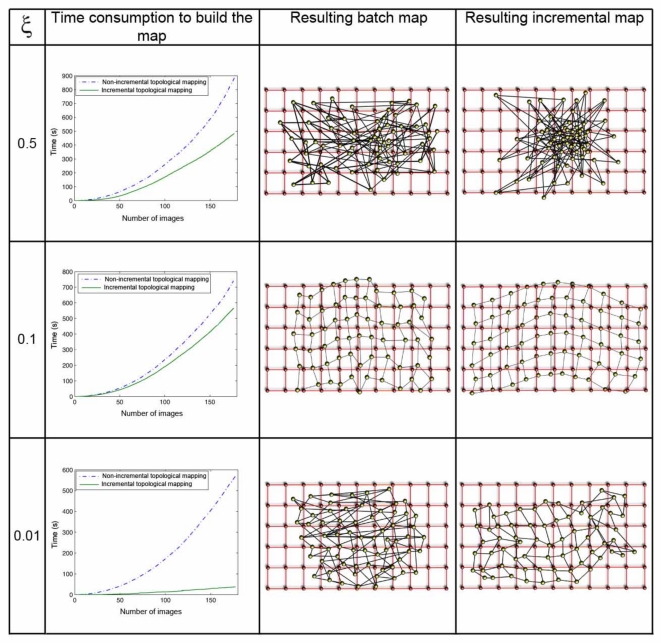
Processing time to build the topological map of *corridor 1* with both methods (column 1) and comparison between the original images grid (small red circles) and the topological map obtained with the batch (column 2) and the incremental (column 3) method method (big yellow circles) when *ξ* = 0.5 (row 1), *ξ* = 0.1 (row 2) and *ξ* = 0.01 (row 3). The grid step is 0.2 m

**Figure 9.**
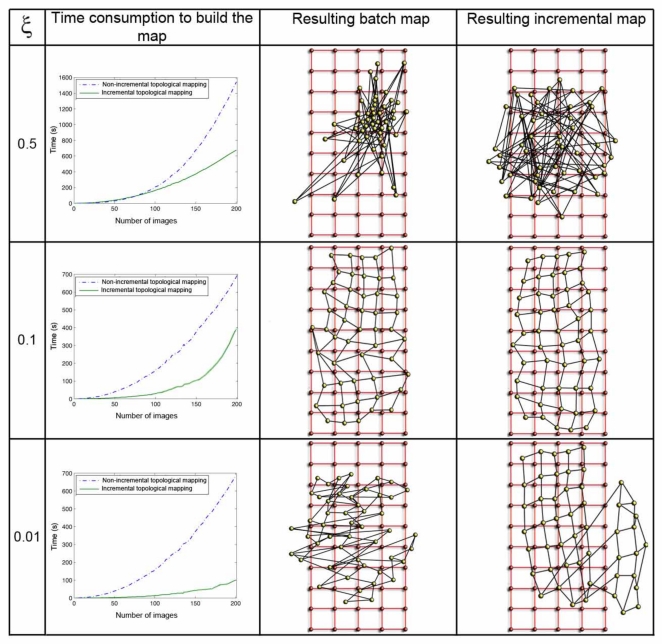
This figure shows the same results than Figure 8 for *laboratory 1*. The grid step is 1 m.

The parameter *ξ* (Equation 8) has a great influence both in the computational cost of the mapping process and in the accuracy of the resulting map. Figure 8 shows clearly this dependence in the environment *corridor 1* (Table 1), with 0.2*m* grid step, and Figure 9 in the environment *laboratory 1*, with 1*m* grid step. In both figures, the first row shows the results when *ξ* = 0.5, the second one when *ξ* = 0.1, and the last one for *ξ* = 0.01. For each value, we show first a graph that presents the time consumption to build the map, depending on the number of images and the method used. Secondly we show the layout of the map built with the batch method, comparing to the original grid and, at last, the map built using the incremental method. In all the cases, using the batch method, we can observe how the necessary time to build the map grows with the number of images since the method has to compute a greater number of forces at each iteration (due to the high number of neighbors each image has). As expected, with the incremental method, the necessary time also grows with the number of images, but to a lesser extent. As *ξ* grows, the computation time also increases, but if a too high or a too low value of *ξ* is taken, the layout of the map built is absolutely different to the layout of the grid where the images were captured. This is due to the fact that *ξ* influences the way the system tends to balance, and it is only reached if we let it freely evolve during the necessary time. This way, we must reach a compromise between the computational cost of the process and the accuracy of the layout obtained to represent the original map.

**Figure 10.**
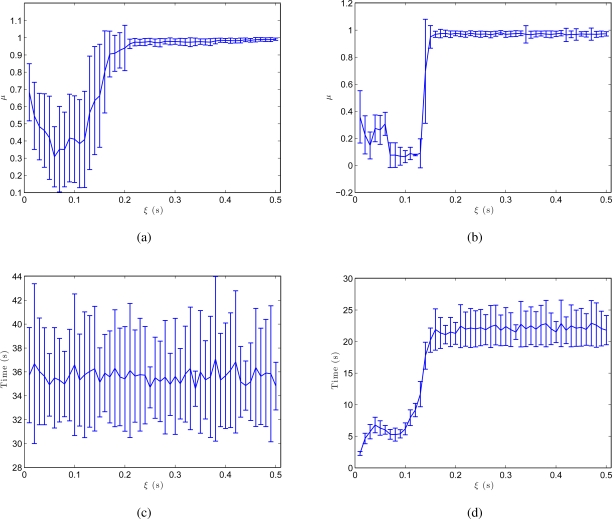
**(a)** Shape difference *μ* versus *ξ* in the batch method and **(b)** in the incremental method. **(c)** Processing time to build the map (for an average map with 50 locations) versus *ξ* in the batch method and **(d)** in the incremental method.

The results shown in Figure 10 (a,b) confirm these conclusions. These figures are obtained from the results of all the environments in Table 1 and show the shape difference *μ* between the resulting map and the initial grid and the processing time until this map is built. As we expected, both in the batch and in the incremental approach, *μ* reaches a minimum for an intermediate value of *ξ*. This is the value that produces the map whose layout of particles is the most similar to the real layout. The incremental method produces a more optimal minimum. As far as processing time is concerned, Figure 10 (c,d) show how this time does not depend on *ξ* in the batch method (only the number of images in the system makes this time to vary) and in the incremental method, a local minimum is reached almost simultaneously with the minimum in the shape factor.

**Figure 11.**
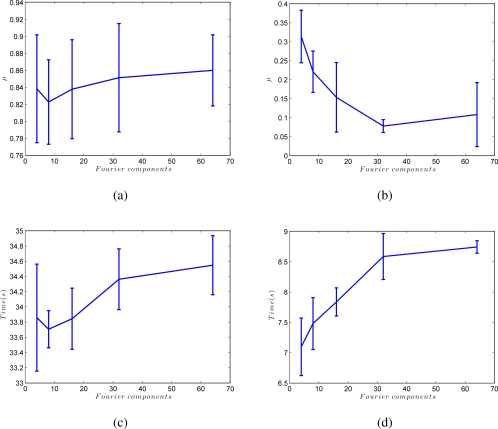
**(a)** Shape difference *μ* versus *k* (number of Fourier components per row in the Fourier signature) in the batch method and **(b)** in the incremental method. **(c)** Processing time to build the map (for an average map with 50 locations) versus *k* in the batch method and **(d)** in the incremental method.

At last, Figure 11 shows the influence of the number of Fourier components we retain per row to build the Fourier signature, *k*. Figure 11 (a,b) shows how the batch method is only slightly affected by the value of *k*, but in the incremental approach, a compromise must be reached between processing time and accuracy of the resulting map when choosing the value of *k*. This figure has also been obtained from the results of all the environments in Table 1.

### 5.2. Monte Carlo localization experiments

**Figure 12.**
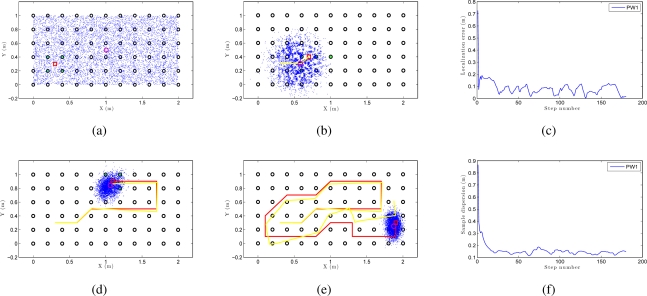
**(a)**, **(b)**, **(d)** and **(e)** are an example of some intermediate steps in a global Monte Carlo localization process using the PW1 and 8000 particles in *corridor 1*, **(c)** error in location for the previous experiment and **(f)** dispersion of the particles for the experiment.

In this section we show separately the results obtained for the *corridor 1* and the *laboratory 1* environments (Table 1). First we present the results with the *corridor 1*. This map is formed by a set of images placed in a grid with a resolution of 0.2*m*. We have taken the omnidirectional images manually so the coordinates of these points are known. The position of this set of images of the map is represented with black circles in Figure 12(a,b,d and e). Next, some sets of images have been taken along some trajectories in the environment every 0, 1*m*. In order to obtain a robust result, we have tested different types of trajectories (a trajectory is a set of omnidirectional images acquired on consecutive points along the environment). Each trajectory has different number of images and also the path is different.

We can see an example of global localization using the method PW1 and 8,000 particles with our Monte Carlo algorithm in Figure 12. As it is a global localization we can see in Figure 12 (a) that the particles presents a uniform distribution when the experiments begin. In the sequence presented in Figure 12 (a,b,d and e) we can observe how the particles concentrate near the true pose. Note that the trajectory does not coincide exactly with the position of any of the images in the map, so it is not necessary to place the vision system exactly on a point in the map to localize precisely the image. In these figures, the ground truth trajectory is shown in red, the path estimated with the odometry data in yellow and the set particles in blue. Figure 12 (c) presents the error in position at each iteration step. The figure shows that the error in location is gradually reduced and so does the dispersion of particles, as it can be observed in Figure 12 (f). Both variables get an acceptable value, under 0.2m (separation between reference images). In this sample process, the length of the route is 180 images (18 m) and the step time is around 0.3 seconds per iteration in our system. This way, this process can be carried out online.

**Figure 13.**
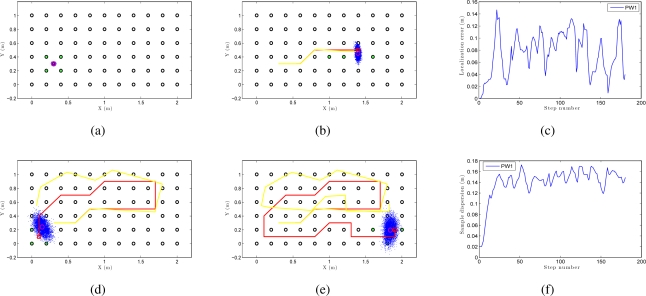
**(a)**, **(b)**, **(d)** and **(e)** are an example of some intermediate steps in a local Monte Carlo localization process using the PW1 and 8000 particles in *corridor 1*, **(c)** error in location for the previous experiment and **(f)** dispersion of the particles for the experiment.

Figure 13 is an example of local localization or tracking using the method PW1 and 8000 particles with our Monte Carlo algorithm. In Figure 13(a) the particles present a gaussian distribution centered on the initial position of the camera when the experiments begin. In the sequence presented in Figure 13 (a,b,d and e) we can observe how the particles disperse near the true pose of the camera. In this experiment, as in the global localization experiment, the trajectory of the camera does not coincide exactly with the position of any of the images in the map. In consequence, the localization can be performed without having to place the robot exactly on a landmark. Figure 13 (c) presents the error in position at each iteration step and Figure 13 (f) presents the dispersion of particles at each iteration.

**Figure 14.**
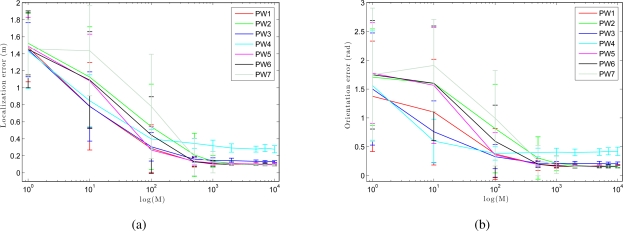
**(a)** Trajectory average error in position and **(b)** orientation versus the number of particles M when using different weighting methods in *corridor 1*.

To compare the performance of our *Monte Carlo localization algorithm* under our seven different types of weighting, we have carried out a series of experiments of global localization in which we have obtained the trajectory average error in position and orientation of the robot depending on the number of particles *M*. For this experiment we have used nine particles population: 1, 10, 100, 500, 1,000, 2,000, 5,000, 7,000 and 10,000 particles. As shown in Figure 14, as we increase the number of particles, the error decreases both in location and orientation. When we use the method PW3 we get nice localization results even with a low number of particles, comparing to the others weight methods.

**Figure 15.**
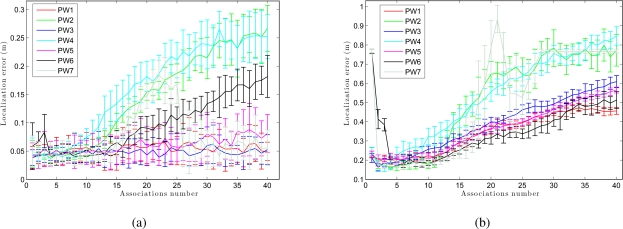
Trajectory average error in position versus associations number for all methods using 8,000 particles. **(a)** Tracking and **(b)** global localization in *corridor 1*.

We have separated the global localization from the tracking to compare the weight methods with respect to the number of associations. Figure 15 (a) presents the error in tracking when varying the number of associations *B*. As shown in Figure 15 (a), although the number of associations is low, the error in localization remains small. As the number of associations increases, the error in the location decreases in the sum-of-gaussian methods, but increases rapidly in the product of gaussian methods (PW2 and PW4). When we multiply two gaussians we get a gaussian with variance lower than the minimum variance of both. Therefore, as the number of associations grows, the weighting of particles becomes more restrictive. On the other hand, Figure 15 (b) presents the results in global localization. When the number of associations is increased, the error in the location grows quicker comparing to the case of tracking (Figure 15 (a)). Moreover, we observe that the last 2 methods (PW6 and PW7) require a minimum number of associations to work properly.

The second map we have tested is the *laboratory 1* environment, formed by a set of images placed in a grid with a resolution of 1 m (Table 1). We have taken the omnidirectional images manually so the coordinates of these points are known. Next, some sets of images have been taken along some trajectories in the environment every 0,5 m. In order to obtain a robust result, we have tested different types of trajectories. Each trajectory has got a different number of images captured and the path is different too. The next figures show the results obtained using this new map.

**Figure 16.**
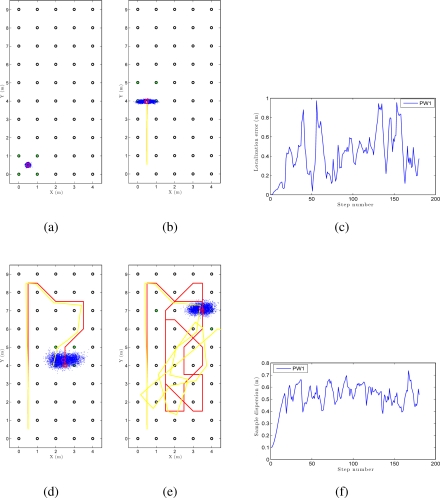
**(a)**, **(b)**, **(d)** and **(e)** are an example of some intermediate steps in a local Monte Carlo localization process using the PW1 and 8000 particles in *laboratory 1*, **(c)** error in location for the previous experiment and **(f)** error in orientation for the experiment.

**Figure 17.**
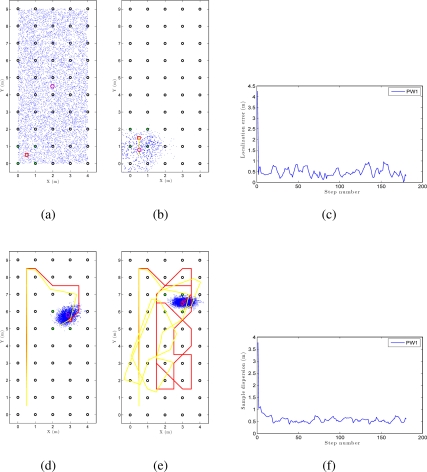
**(a)**, **(b)**, **(d)** and **(e)** are an example of some intermediate steps in a global Monte Carlo localization process using the PW1 and 8,000 particles in *laboratory 1*, **(c)** error in location for the previous experiment and **(f)** error in orientation for the experiment.

Both in the case of global localization (Figure 17) and in the case of local localization or tracking (Figure 16) we can observe as the localization error and the sample dispersion tend to an acceptable value around the separation between reference images (0.5*m*) when we use PW1 and 8,000 particles.

**Figure 18.**
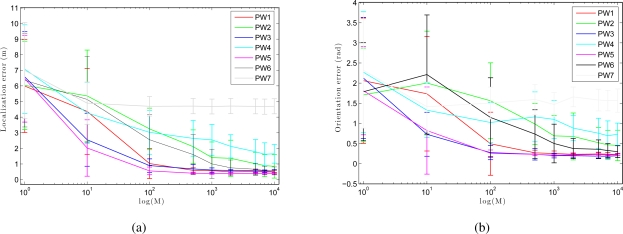
**(a)** Trajectory average error in position and **(b)** orientation versus the number of particles M when using different weighting methods in *laboratory 1*.

Figure 18 shows the trajectory average error and the orientation error when we use different number of particles (*M*). As in the previous environment, the methods PW1, PW3 and PW5 present relatively good results when a low number of particles is taken. The methods PW3 and PW5 present a better behavior for any number of particles.

**Figure 19.**
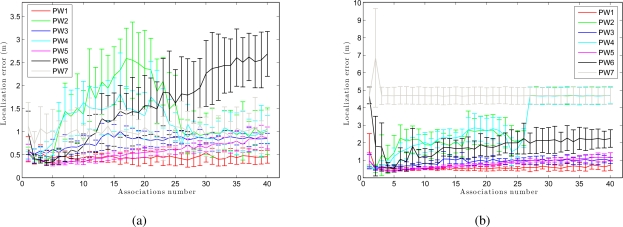
Trajectory average error in position versus associations number for all methods using 8.000 particles. **(a)** Tracking and **(b)** global localization in *laboratory 1*.

At last, Figure 19 shows the trajectory average error in a tracking and in a global localization when we use different associations number. From this figure we observe that PW1, PW3 and PW5 have a more stable behavior at any associations number although they present a lower error when the number of associations is relatively low. The method PW7 does not work properly in this environment and at last, PW2 and PW4 present, in general, a worse result.

## 6. Conclusions and Future Work

In this paper we have studied the applicability of the approaches based on the global appearance of omnidirectional images in topological mapping and localization tasks. The main contributions of the paper include the development of an approach to build a topological map of the environment in an incremental way, the study of the influence of the parameters of the process in the layout of the resulting map and in the processing time, the analysis of an appearance-based Monte Carlo localization using omnidirectional images, the comparison between different weighting methods both in the case of local and global localization and the influence of the Fourier signature descriptor in the mapping and localization process.

All the experiments have been carried out with a set of omnidirectional images captured by a catadioptric system mounted on the mobile platform. Each scene is first filtered to avoid lighting dependance and then it is described through a Fourier-based signature that presents a good performance in terms of amount of memory and processing time, and it is also invariant to ground-plane rotations and an inherently incremental method.

We present a methodology to build incremental topological maps. As shown in the results, when the parameters of this system are correctly tuned, accurate results can be obtained, maintaining a reasonable computational cost. The incremental method clearly outperforms the batch one. However, in this method, it is necessary to arrive to a compromise between accuracy and processing time when choosing the number of Fourier components in the descriptor.

On the other hand, we have presented a Monte Carlo localization method using omnidirectional images and we have evaluated the performance of different weighting methods in the case of local and global localization, finding different behaviors. We have also evaluated the influence of the descriptor in the localization by varying the number of possible associations. Our system is able to estimate the position of the robot in the case of unknown initial position and it is able to track the position of the robot while moving. We proved how the accuracy of the methods varies with the type of weight and the number of particles and associations. In the evaluated methods, as we increase the number of particles in the system, the average error of localization decreases rapidly. With respect to the orientation, we obtain similar results. We have proved the strong dependence between the number of associations and the type of method used. Also, it is possible to correct the weighting of the particles by combining a physical system of forces with a Gaussian weight (PW6).

The experimental section includes results both for a structured environment, which presents repetitive visual aspects, and an unstructured one (a corridor and a laboratory, respectively). In both cases, we have obtained relatively good results in the mapping and in the localization processes.

We are working now in the fusion of these two systems to build a topological SLAM approach (Simultaneous Localization and Map Building) using just the global appearance of omnidirectional images, and different map topologies.

## Figures and Tables

**Table 1.** Relevant parameters of the image sets used in the mapping experiments.

**Environment**	**Size (Images)**	**Resolution**	**Grid step**	**Environment size**
**Laboratory 1**	5 × 10	56 × 256 pixels	1 m	6 × 11 m
**Laboratory 2**	10 × 15	56 × 256 pixels	0.3 m	3.5 × 4.5 m
**Office**	6 × 8	56 × 256 pixels	0.5 m	3.5 × 4.5 m
**Hall**	12 × 9	56 × 256 pixels	0.1 m	1.3 × 1 m
**Corridor 1**	11 × 6	56 × 256 pixels	0.2 m	2.4 × 1.5 m
**Corridor 2**	35 × 10	56 × 256 pixels	0.1 m	3.6 × 1.1 m

## References

[1] Paya L, Fernandez L, Reinoso O, Gil A, Ubeda D (2009). Appearance-based Dense Maps Creation. Comparison of Compression Techniques with Panoramic Images.

[2] Winters N, Gaspar J, Lacey G, Santos-Victor J (2000). Omni-directional Vision for Robot Navigation.

[3] Se S, Lowe D, Little J (2010). Vision-based Mobile Robot Localization and Mapping using Scale-Invariant Features.

[4] Lowe D (2004). Distinctive Image Features from Scale-Invariant Keypoints. Int J Comput Vision.

[5] Lingua A, Marenchino D, Nex F (2009). Performance Analysis of the SIFT Operator for Automatic Feature Extraction and Matching in Photogrammetric Applications. Sensors.

[6] Valgren C, Lilienthal A (2010). SIFT, SURF and Seasons: Appearance-based Long-term Localization in Outdoor Environments. Robot Auton Systems.

[7] Murillo AC, Guerrero JJ, Sagüés C (2007). SURF Features for Efficient Robot Localization with Omnidirectional Images.

[8] Bay H, Tuytelaars T, Van Gool L (2006). SURF: Speeded Up Robust Features.

[9] Menegatti E, Maeda T, Ishiguro H (2004). Image-based Memory for Robot Navigation Using Properties of Omnidirectional Images. Robot Auton Systems.

[10] Menegatti E, Zocaratto M, Pagello E, Ishiguro H (2004). Image-based Monte Carlo Localisation with Omnidirectional Images. Robot Auton Systems.

[11] Kröse B, Bunschoten R, Hagen ST, Terwijn B, Vlassis N (2004). Environment Modeling and Localization from an Omnidirectional Vision System. IEEE Robotics Autom Mag.

[12] Kirby M (2001). Geometric Data Analysis.

[13] Moravec H, Elfes A (1985). High Resolution Maps from Wide Angle Sonar.

[14] Collins T, Collins J, Ryan C (2007). Occupancy Grid Mapping: An Empirical Evaluation.

[15] Gil A, Reinoso O, Ballesta M, Juliá M, Payá L (2010). Estimation of Visual Maps with a Robot Network Equipped with Vision Sensors. Sensors.

[16] Werner F, Maire F, Sitte J (2009). Topological SLAM Using Fast Vision Techniques.

[17] Valgren C, Lilienthal A (2007). SIFT, SURF and Seasons: Long-term Outdoor Localization Using Local Features.

[18] Stimec A, Jogan M, Leonardis A (2008). Unsupervised Learning of a Hierarchy of Topological Maps Using Omnidirectional Images. IJPRAI.

[19] Tully S, Kantor G, Choset H, Werner F (2009). A Multi-hypothesis Topological SLAM Approach for Loop Closing on Edge-ordered Graphs.

[20] Angeli A, Doncieux S, Meyer J, Filliat D (2009). Visual Topological SLAM and Global Localization.

[21] Thrun S, Fox D, Burgard W, Dellaert F (2000). Robust Monte Carlo Localization for Mobile Robots. Artif Intell.

[22] Thrun S, Burgard W, Fox D (2000). A Real-Time Algorithm for Mobile Robot Mapping With Applications to Multi-Robot and 3D Mapping. Proceedings of the IEEE Intnational Conferences on Robotics & Automation (ICRA).

[23] Dellaert F, Fox D, Burgard W, Thrun S (1999). Monte Carlo Localization for Mobile Robots.

[24] Gil A, Reinoso O, Vicente MA, Fernández C, Payá L (2005). Monte Carlo Localization Using SIFT Features. Lect Notes Comput Sci.

[25] Pizarro D, Mazo M, Santiso E, Marron M, Jimenez D, Cobreces S, Losada C (2010). Localization of Mobile Robots Using Odometry and an External Vision Sensor. Sensors.

[26] Linåker F, Ishikawa M (2006). Real-time Appearance-based Monte Carlo Localization. Robot Auton Systems.

[27] Jogan M, Leonardis A (2000). Robust Localization Using Eigenspace of Spinning-Images.

[28] Rossi F, Ranganathan A, Dellaert F, Menegatti E (2008). Toward Topological Localization with Spherical Fourier Transform and Uncalibrated Camera.

[29] Adini Y, Moses I, Ullman S (1997). Face Recognition: The Problem of Compensating for Changes in Illumination Direction. IEEE Trans Robot Automat.

[30] Murase H, Nayar S (1994). Illumination Planning for Object Recognition Using Parametric Eigenspaces. IEEE Trans Pattern Anal Mach Intell.

[31] de Araújo VP, Maia RD, D’Angelo MFSV, D’Angelo GNR (2006). Automatic Plate Detection Using Genetic Algorithm. Proceedings of the 6th WSEAS International Conference on Signal, Speech and Image Processing, SSIP’06.

[32] Fernandez L, Paya L, Reinoso O, Gil A, Julia M, Ballesta M (2010). Robust Methods for Robot Localization Under Changing Illumination Conditions. Comparison of Different Filtering Techniques.

[33] Gonzalez RC, Woods RE (1992). Digital Image Processing.

[34] Seber G (1984). Multivariate Observations.

[35] Fox D, Burgard W, Thrun S (1999). Markov Localization for Mobile Robots in Dynamic Environments. JAIR.

[36] Smith AFM, Gelfand AE (1992). Bayesian Statistics without Tears: A Sampling-Resampling Perspective. Amer Statist.

[37] Rubin DB (1988). Using the SIR Algorithm to Simulate Posterior Distributions.

